# Attrition from care and its predictors among women exposed to dolutegravir- and efavirenz-based first-line antiretroviral therapy in Ethiopia: a before-and-after study

**DOI:** 10.3389/fpubh.2024.1385441

**Published:** 2024-07-02

**Authors:** Wolde Facha, Takele Tadesse, Eskinder Wolka, Ayalew Astatkie

**Affiliations:** ^1^Department of Epidemiology and Biostatistics, School of Public Health, College of Health Sciences and Medicine, Wolaita Sodo University, Wolaita Sodo, Ethiopia; ^2^School of Public Health, College of Medicine and Health Sciences, Hawassa University, Hawassa, Ethiopia

**Keywords:** attrition, lost to follow-up, retention, dolutegravir, efavirenz, Ethiopia

## Abstract

**Background:**

The effect of dolutegravir (DTG)-based regimens on reducing attrition from care among women enrolled in the prevention of mother-to-child transmission (PMTCT) care program is unknown. Therefore, this study aimed to compare the incidence of attrition among women exposed to DTG-based with those exposed to efavirenz (EFV)-based first-line antiretroviral therapy (ART) in Ethiopia.

**Methods:**

An uncontrolled before-and-after study was conducted involving 932 women (with 466 on EFV-based and 466 on DTG-based regimens) who were enrolled in the PMTCT care program from September 2015 to February 2023. The outcome variable was attrition (i.e., maternal death or loss to follow-up before their infants' final HIV status was determined). A Kaplan–Meier estimator was employed to estimate the probability of attrition. The Cox proportional hazards regression model was fitted to identify predictor variables. The adjusted hazard ratio (aHR) with the corresponding 95% confidence interval (CI) was calculated to examine the risk difference in the comparison groups.

**Results:**

The cumulative incidence of attrition among women was 5.2% (3.0% for those placed in the DTG-based regimen arm and 7.3% for those placed in the EFV-based regimen arm). Women on DTG-based regimens had a 57% (aHR: 0.43; 95% CI: 0.23–0.80) lower risk of attrition from care compared to those on EFV-based regimens. Women who delivered their infants at home (aHR: 2.35; 95% CI: 1.14–4.85), had poor/fair adherence (aHR: 3.23; 95% CI: 1.62–6.45), had unsuppressed/unknown viral load status (aHR: 2.61; 95% CI: 1.42–4.79), and did not disclose their status to partners (aHR: 2.56; 95% CI: 1.34–4.92) had a higher risk of attrition from PMTCT care compared to their counterparts.

**Conclusion:**

The cumulative incidence of attrition among women receiving PMTCT care is optimal. In addition, the risk of attrition among women receiving DTG-based regimens is lower than that among women receiving EFV-based regimens. Thus, DTG-based first-line ART regimen supplementation should be sustained to achieve a national retention target of 95% and above.

## 1 Introduction

Mother-to-child transmission (MTCT) of the human immunodeficiency virus (HIV) is the most common cause of infection in children (1). Globally, at the end of 2022, approximately 1.5 million children were living with HIV and 130,000 acquired the virus (2). Antiretroviral therapy (ART) and other interventions can reduce the risk of transmission to <5% in breastfeeding women and to <2% in non-breastfeeding women (3).

Ethiopia adopted the World Health Organization's (WHO) Option B plus recommendations as the preferred strategy for the prevention of mother-to-child transmission (PMTCT) of HIV in 2013 (4). Option B plus involves lifelong ART for all HIV-infected pregnant and breastfeeding women, irrespective of their immunological status and WHO clinical staging (3). This strategy is designed to deliver high-quality service to improve the quality of maternal life and reduce the incidence of MTCT of HIV among exposed infants (5). In 2022, 82% of pregnant women with HIV had access to ART to prevent the transmission of the virus to their infants during pregnancy and childbirth and to protect their own health (6). However, the effectiveness and quality of Option B plus depend on retaining women throughout the continuum of care during pregnancy and breastfeeding (7). In addition, attrition poses a major challenge in the PMTCT program aimed at ending Acquired Immune Deficiency Syndrome (AIDS) as a public health threat by 2030 (8).

The WHO updated different treatment guidelines, and Ethiopia adopted the WHO protocol to prevent new pediatric HIV infections and improve the survival of mothers and their infants (3, 5, 9). Accordingly, as of the end of 2018, a dolutegravir (DTG)-based regimen is recommended as the first-line treatment for people living with HIV, and this recommendation expanded to include pregnant and breastfeeding women as of July 2019 (10, 11). However, failure to retain women in care hinders the benefits of the drug in reducing the MTCT of HIV.

According to the targets set by the United Nations Program on HIV/AIDS (UNAIDS), Ethiopia has aimed to achieve a 95% retention rate (i.e., patients being alive and receiving ART) in care since 2020 (8, 12). However, various studies in Africa, including Ethiopia, indicate that the retention rate is below this global target of 95% (13–18). Previous studies have focused on the magnitude and risk factors of attrition among women on efavirenz (EFV)-based regimens (13, 14, 16, 17, 19, 20). Despite the preference for DTG-based regimens due to their high efficacy, better tolerability, high genetic resistance barrier, low cost, rapid viral suppression, and fewer side effects compared to other antiretroviral drugs, their effectiveness in retaining women in PMTCT care over the previously used EFV-based regimens has not been well investigated (3, 11, 21). Therefore, this study aims to compare the incidence of attrition and its risk factors among women receiving DTG-based with those receiving EFV-based first-line ART in Ethiopia.

## 2 Materials and methods

### 2.1 Study design and period

An uncontrolled before-and-after study was conducted among women enrolled in the PMTCT care program from September 2015 to February 2023. Data were retrieved from the records between 10 March 2023 and 25 May 2023.

### 2.2 Study setting and population

The study was conducted in two regions of Ethiopia: Central Ethiopia and South Ethiopia. Currently, these regions consist of 140 health facilities (49 hospitals and 91 health centers) that provide ART and PMTCT services to 1,236 women in the PMTCT care program (675 in South Ethiopia and 561 in Central Ethiopia). Among 72 facilities that have been providing both PMTCT and ART services since 2015 when the study started, 34 facilities (20 hospitals and 14 health centers) were selected. The source population for the unexposed (before) group comprised all pregnant and breastfeeding women on EFV-based first-line ART, while that for the exposed (after) group consisted of all pregnant and breastfeeding women on DTG-based first-line ART in Ethiopia. All eligible women who enrolled in PMTCT care from September 2015 onward and received only EFV-based regimens until discharge were recruited for the unexposed (before) group, whereas all eligible women who received only DTG-based regimens during the entire PMTCT period until discharge were recruited for the exposed (after) group.

### 2.3 Sample size determination and sampling technique

The sample size was calculated using the double population proportion formula with G Power statistical software version 3.1.9.7. A significance level (alpha) of 5%, a power of 80%, an 84.6% retention rate in care among the unexposed group (17), and a ratio of unexposed to exposed of 1 were considered. We used an effect size of 0.2 (which is a small effect size that is conventionally used since there is no evidence supporting the magnitude of attrition among women exposed to DTG-based regimens). After considering 20% of missing data, the total sample size was 932 (466 for DTG-based regimens and 466 for EFV-based regimens). The final sample size is allocated proportionally to the number of pregnant and breastfeeding women enrolled in PMTCT care across all 34 health facilities. All eligible women in the selected facilities were included in the study until the expected sample size was reached in both groups.

### 2.4 Operational definitions

**Exposed group** refers to pregnant and breastfeeding women who have received DTG-based first-line ART until discharge from the PMTCT program.

**Linked to ART** refers to a woman who has transferred from Option B plus PMTCT care to the ART unit for continuity of care after her infant's final HIV status is determined.

**Lost to follow-up (LTFU)** refers to a woman who has not been seen within 28 days of the last scheduled clinic appointment and is not registered as dead or transferred to another clinic before the infant's final HIV status is determined (22).

**Attrition** refers to a woman enrolled in Option B plus PMTCT care who either died or is LTFU before the infant's final HIV status is determined (9).

**Retention** refers to a situation whereby a woman who is alive and on Option B plus PMTCT care until her infant's final HIV status is determined (9).

**Non-retention** refers to a situation in which women are not in the PMTCT program due to death, LTFU, or linkage to ART.

**Transfer out** refers to a woman who started ART in one facility but transferred to another facility for continuity of care with a standard transfer-out form (3).

**Unexposed group** refers to pregnant and breastfeeding women who are on EFV-based first-line ART until discharge from the PMTCT program.

### 2.5 Study variables

The outcome variable was maternal attrition from care among women on Option B plus PMTCT care. Women who remained in care until their infants' final HIV status was determined were categorized as censored, whereas those who died or were LTFU before their infants' final HIV status was determined were categorized as attrition, i.e., the event of interest. The exposure variable was the ART regimen the mother was receiving. Covariates in the present study were maternal sociodemographic, obstetric, and drug- and clinical-related variables. Mothers' ART adherence was categorized as poor, fair, or good (3, 22, 23). A viral load measurement not detected or below 50 copies/ml throughout the follow-up period is indicated as a suppressed viral load status, whereas a viral load measurement above 50 copies/ml at any time of the follow-up period is indicated as an unsuppressed viral load status (3, 22, 24). Details of the measurement of the variables are provided elsewhere (25).

### 2.6 Data management and analysis

Data were retrieved from the PMTCT registration book, Smart Care (a computer-based data registry found at the ART unit of respective facilities), and women's hospital records, which included the intake forms and follow-up cards. The data were collected using Open Data Kit (ODK) version 2.4 and then exported to Stata 14.0 (StataCorp, College Station, Texas, U.S.A.) for analysis. The event (i.e., death or LTFU) was coded as 1, and the censor (i.e., retention in care until the infant's final outcome was determined) was coded as 0. The Kaplan–Meier estimates were used to calculate the probabilities of attrition at the 6^th^, 12^th^, and 18^th^ months of enrollment. A log-rank test was used to determine the difference in survival experiences between the two categories. The proportional hazard assumption was assessed using the log–log plot. In addition, the Cox–Snell residual and global tests were conducted, and the model was found to be fit (*p* = 0.2669). A bivariable Cox regression analysis was conducted on covariates that fulfilled the proportional hazards assumption. Covariates with a *p*-value of < 0.25 in the unadjusted analyses were selected as candidate variables for multivariate analysis. Then, a multivariable Cox proportional hazards regression model was fitted to identify the effect of DTG-based regimens and other predictor variables on attrition from care. The adjusted hazard ratio (aHR) with 95% confidence interval (CI) was computed, and all predictors that were associated with the outcome variable with a *p*-value of ≤ 0.05 were declared as a significant predictor of the outcome variable. Stratification was performed for occupation, facility type, CD4 count, and enrollment type that violated the proportional hazards assumption. However, there was no significant difference in the risk of attrition among women on DTG-based regimens.

## 3 Results

### 3.1 Sociodemographic characteristics

A total of 956 women's documents were reviewed and 932 study subjects were included in our study, achieving a response rate of 97.5%. In this study, 44.4% of women in the DTG-based regimen arm and 41.4% of women in the EFV-based regimen arm were over the age of 30 years. Additionally, 19.1% of women in the DTG-based regimen arm and 16.9% of women in the EFV-based regimen arm had high-risk occupations. On the other hand, 35.4% of women in the DTG-based regimen arm and 40.1% in the EFV-based regimen arm did not have a formal education (Table 1).

**Table 1:** Sociodemographic characteristics of the mothers.

**Variables**	**DTG-based regimen arm (*n =* 466)**	**EFV-based regimen arm (*n =* 466)**	***P*-value^dag^**
**Age (in years)**			
15–29	259 (55.6)	273 (58.6)	0.354
30–45	207 (44.4)	193 (41.4)	
**Residence**			
Rural	120 (25.7)	137 (29.4)	0.213
Urban	346 (74.3)	329 (70.6)	
**Occupation**			
Low risk^*^	377 (80.9)	387 (83.1)	0.394
High risk^**^	89 (19.1)	79 (16.9)	
**Educational status**			
Formal	301 (64.6)	279 (59.9)	0.137
Not formal	165 (35.4)	187 (40.1)	
**Marital status**			
Married	404 (86.7)	405 (86.9)	0.923
Divorced/widowed	62 (13.3)	61 (13.1)	

^*^Low risk, housewife, employee, merchant, students; ^**^High risk, commercial sex workers and daily laborers † Pearson's chi-squared test.

### 3.2 Obstetric characteristics

In our study, 85.2% of women in the DTG-based regimen arm and 90.1% of women in the EFV-based regimen arm had attended antenatal care during their pregnancy. However, 7.3% of women in the DTG-based regimen arm and 9.0% of women in the EFV-based regimen arm delivered their infants at home (Table 2).

**Table 2:** Obstetric characteristics of the mothers.

**Variables**	**DTG-based regimen arm (*n =* 466)**	**EFV-based regimen arm (*n =* 466)**	***P*-value^†^**
**Attended ANC** ^*^			
Yes	397 (85.2)	420 (90.1)	0.022
No	69 (14.8)	46 (9.9)	
**Tested for syphilis**			
Yes	379 (81.3)	374 (80.3)	0.173
No	87 (18.7)	92 (19.7)	
**Place of delivery**			
Health facility	432 (92.7)	424 (91.0)	0.338
Home	34 (7.3)	42 (9.0)	

^*^ANC, antenatal care ^†^Pearson's chi-squared test.

### 3.3 Drug- and clinical-related characteristics

In this study, 48 (5.2%) women (3.0% in the DTG-based regimen arm and 7.3% in the EFV-based regimen arm) died or were LTFU from PMTCT care. In addition, 30.3% of women in the DTG-based regimen arm and 33.1% of women in the EFV-based regimen arm were newly enrolled in PMTCT care; 6.4% of them in the DTG-based regimen arm and 9.9% in the EFV-based regimen arm started ART during their delivery or breastfeeding period. Moreover, 6.4% of women in the DTG-based regimen arm and 8.4% in the EFV-based regimen arm had poor/fair adherence to ART during the PMTCT period. Furthermore, 16.7% of women in the DTG-based regimen arm and 15.0% of women in the EFV-based regimen arm did not disclose their HIV status to their partners. The median [interquartile range (IQR)] maternal PMTCT duration was 21.7 (18.7–24.3) months in the DTG-based regimen arm and 21.9 (18.6–24.7) months in the EFV-based regimen arm (Table 3).

**Table 3:** Drug- and clinical-related characteristics of women.

**Variables**	**DTG-based regimen arm (*n =* 466)**	**EFV-based regimen arm (*n =* 466)**	***P*-value^†^**
**Maternal outcome**			
Linked to ART^*^	452 (97.0)	432 (92.7)	0.003
Died/LTFU^**^	14 (3.0)	34 (7.3)	
**Types of facility**			
Health center	168 (36.1)	127 (27.3)	0.004
Hospital	298 (63.9)	339 (72.7)	
**Enrollment type**			
Known	325 (69.7)	312 (66.9)	0.360
New	141 (30.3)	154 (33.1)	
**When ART started**			
Before delivery	436 (93.6)	420 (90.1)	0.055
During delivery/breastfeeding	30 (6.4)	46 (9.9)	
**Adherence status**			
Good	436 (93.6)	427 (91.6)	0.260
Poor/fair	30 (6.4)	39 (8.4)	
**Viral load status**			
Suppressed	400 (85.8)	363 (77.9)	0.002
Unsuppressed/unknown	66 (14.2)	103 (22.1)	
**Partner HIV status**			
Negative	150 (32.2)	121 (26.0)	0.036
Positive/unknown	316 (67.8)	345 (70.0)	
**Disclosure status**			
Yes	388 (83.3)	396 (85.0)	0.473
No	78 (16.7)	70 (15.0)	
**WHO stage**			
Stage 1	450 (96.6)	446 (95.7)	0.497
Stage >=2	16 (3.4)	20 (4.3)	
**Duration in PMTCT care (in months)**			
Median(IQR)	21.7 (18.7–24.3)	21.9 (18.6–24.7)	0.407

^†^Pearson's chi-squared test.

### 3.4 Survival estimates

**Figure 1:**
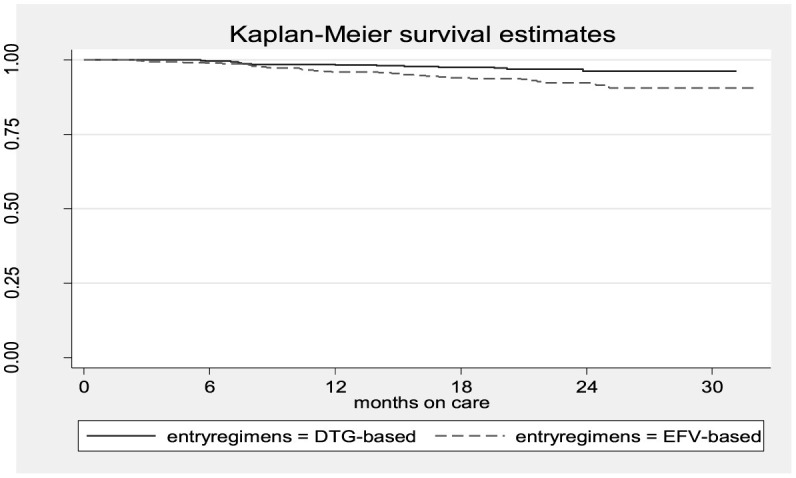
The Kaplan–Meier survival estimates of women who received DTG-based vs. those who received EFV-based first-line ART.

The Kaplan–Meier survival estimates indicated that women in the DTG-based regimen arm had a higher survival rate (retention in care) than those in the EFV-based regimen arm (Figure 1).

**Figure 2:**
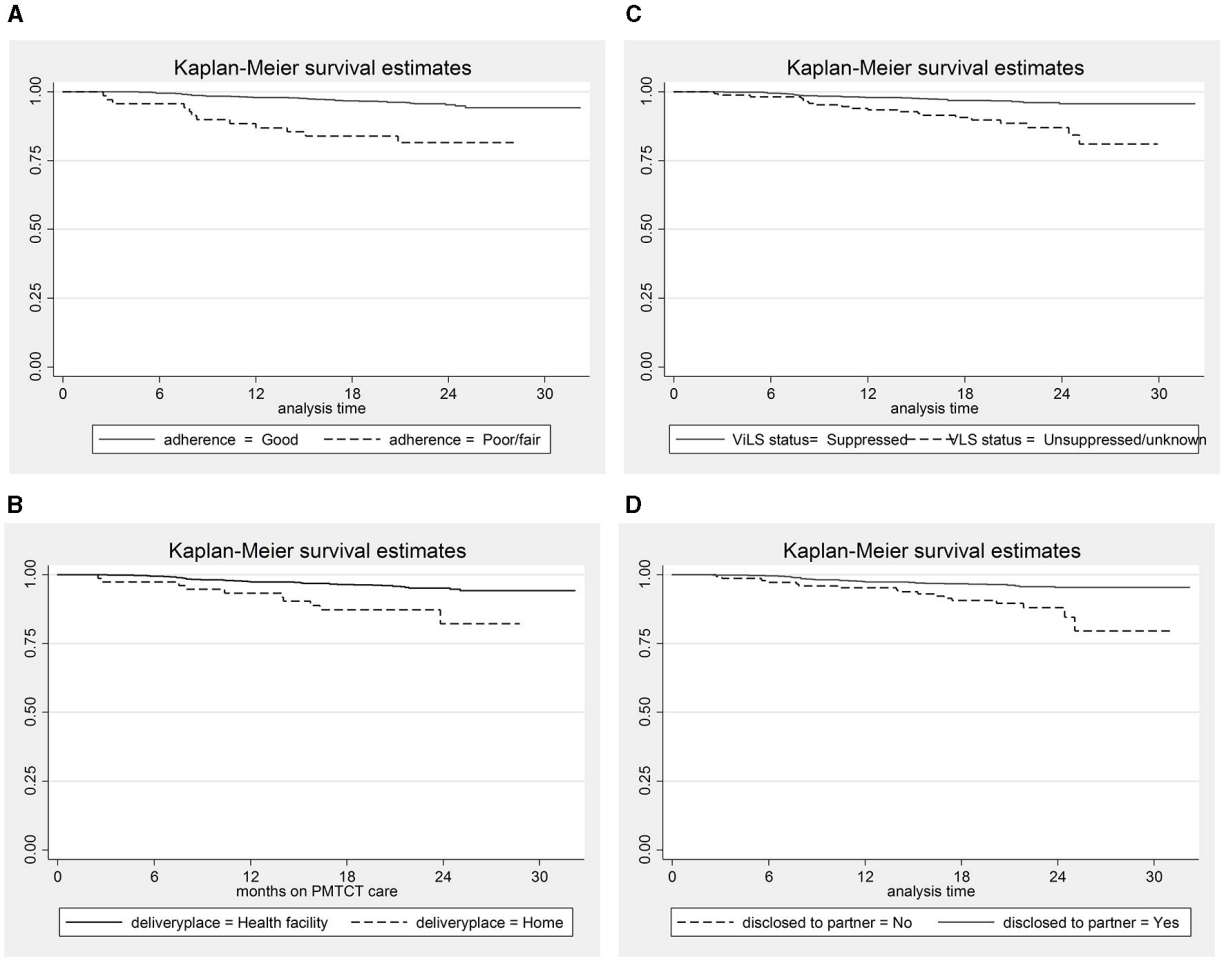
The Kaplan–Meier survival estimates for predictor variables. **(A)** Adherence status; **(B)** place of delivery; **(C)** Viral load suppression status; **(D)** Disclosure status.

In addition, women who did not disclose their HIV status to their sexual partners, had unsuppressed/unknown viral load status, had poor/fair adherence to ART, and delivered their infants at home had a lower survival rate than their counterparts (Figure 2).

### 3.5 Maternal attrition status

**Figure 3:**
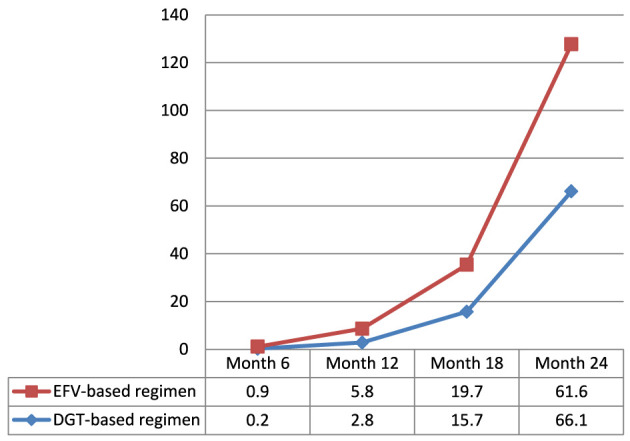
Magnitude of non-retention status in PMTCT care among women exposed to DTG-based compared to those exposed to EFV-based first-line antiretroviral therapy.

The cumulative incidence of attrition from PMTCT care among women is 5.2% [95% CI: 3.9%−6.8%; (3.0% in the DTG-based regimen arm and 7.3% in the EFV-based regimen arm)]. There was a lower non-retention rate among women in the DTG-based regimen arm compared to those in the EFV-based regimen arm at the 6^th^, 12^th^, and 18^th^ months (Figure 3).

### 3.6 Effect of DTG-based regimens on attrition status

In a multivariable Cox proportional hazards regression analysis, mothers who were on a DTG-based regimen had approximately 57% (aHR: 0.43; 95% CI: 0.23–0.80) lesser hazard of attrition from PMTCT care than those who were on an EFV-based regimen. In addition, mothers who delivered their infants at home were 2.35 times (aHR: 2.35; 95% CI: 1.14–4.85) more likely to experience attrition from PMTCT care than their counterparts. Similarly, those who exhibited poor/fair adherence to ART were 3.23 times (aHR: 3.23; 95% CI: 1.62–6.45) more likely to do so. Mothers who had unsuppressed/unknown viral load status were 2.61 times (aHR: 2.61; 95% CI: 1.42–4.79) more likely to drop out, and those who did not disclose their status to partner were 2.56 times (aHR: 2.56; 95% CI: 1.34–4.92) more likely to experience attrition from PMTCT care than their counterparts (Table 4).

**Table 4:** Effect of DTG-based first-line ART regimens and other covariates on maternal attrition in PMTCT care.

**Variables**	**Maternal attrition status**		**cHR (95% CI)**	**aHR (95% CI)**
	**Yes N**°**(%)**	**No N**°**(%)**		
**PMTCT drug regimens**				
DTG-based	14 (3.0)	452 (97.0)	0.41 (0.22–0.76) ^††^	0.43 (0.23–0.80) ^††^
EFV-based	34 (7.3)	432 (92.7)		
**Education**				
No formal	25 (7.1)	327 (92.9)	1.79 (1.02–3.16) ^††^	1.39 (0.78–2.49)
Formal	23 (4.0)	557 (96.0)	1	1
**Marital status**				
Divorced/widowed	11 (8.9)	112 (91.1)	2.04 (1.04–3.99) ^††^	1.25 (0.60–2.61)
Married	37 (4.6)	772 (95.4)	1	1
**Delivery place**				
Home	10 (13.2)	66 (86.8)	3.36 (1.67–6.75) ^†^	2.35 (1.14–4.85) ^††^
Health facility	38 (4.4)	818 (95.6)	1	1
**Adherence status**				
Poor/fair	12 (17.4)	57 (82.6)	4.54 (2.36–8.73) ^†^	3.23 (1.62–6.45) ^†^
Good	36 (4.2)	827 (95.8)	1	1
**VLS** ^*^ **status**				
Unsuppressed/Unknown	20 (11.8)	149 (88.2)	3.64 (2.05–6.47) ^†^	2.61 (1.42–4.79) ^††^
Suppressed	28 (3.7)	735 (96.3)	1	1
**Disclosed to partner**				
No	17 (11.5)	131 (88.5)	3.15 (1.74–5.69) ^†^	2.56 (1.34–4.92) ^††^
Yes	31 (3.9)	753 (96.1)	1	1
**When started ART** ^**^				
During delivery/BF^***^	7 (9.2)	69 (90.8)	2.46 (1.1–5.5) ^††^	1.08 (0.44–2.65)
Before delivery	41 (4.8)	815 (95.2)	1	1

^*^VLS, viral load suppression; ^**^ART, antiretroviral therapy; ^***^BF, breastfeeding; ^†^*p* < 0.001; ^††^*p* < 0.05.

## 4 Discussion

In this study, women on DTG-based regimens have lower attrition rates than those on EFV-based regimens. Delivery at home, poor/fair maternal adherence to medication, unsuppressed/unknown viral load status, and lack of disclosure to male partners were the identified risk factors for attrition among women in PMTCT care.

Furthermore, the cumulative incidence of attrition among women in PMTCT care was found to be 5.2% (3.0% in the DTG-based regimen and 7.3% in the EFV-based regimen). This finding in this study is lower than the results of the studies conducted in Uganda (14, 15, 26–28), Zimbabwe (16), Cameroon (17), Mozambique (29), and Ethiopia (18, 30–32) and the systematic review and meta-analysis conducted in Africa (13). This difference in the results can be attributed to better tolerability, higher efficacy, and effectiveness of the DTG-based regimen that were included in the study unlike the previous studies that did not include the DTG-based regimen (3, 11, 21). In addition, the differences in operational definitions adopted in the studies may have led to the differing results; these previous studies defined LTFU as women not being seen within 90 days of their last scheduled clinic appointment, whereas our study used 28 days as a cutoff point for LTFU. Women who were not retained in PMTCT care may develop drug resistance, have high viral copies, and transmit HIV to uninfected sexual partners and their infants (3, 22). A mechanism for retaining and tracing patients LTFU should be strengthened in all facilities that provide PMTCT services (9).

In our study, the place of delivery was significantly associated with attrition status. Women who had opted for delivery at home had a 2.35 times higher risk of attrition from PMTCT care compared to those who delivered at health facilities. Women who enrolled in the PMTCT program after delivery faced a higher risk of attrition from care than those who enrolled in the program during pregnancy (33). This finding could be because mothers who enrolled in the PMTCT program after delivery are more likely to have delivery at home and may not have received adequate counseling on the importance of regular clinic visits and the consequences of attrition from care for themselves and their infants (34). Therefore, efforts should focus on mobilizing and creating awareness among pregnant women about the benefits of attending institutional delivery.

Women with poor/fair adherence to ART were found to have a 3.23 times higher risk of attrition from PMTCT care compared to those having good ART adherence. This finding is consistent with the results of studies conducted in different parts of Ethiopia, such as Nekemte (33), East Gojam (35), University of Gondar (36), and Oromia region (37). The possible reasons for poor adherence may include conflicts with religious beliefs, seeking traditional healers, lack of partner support, fear of stigma and discrimination, lack of knowledge on the importance of adherence, and fear of the side effects of drugs, all of which may lead patients to miss their medication (33, 35).

Women who did not disclose their HIV status to their male partners had a 2.56 times higher risk of attrition than those who disclosed it. This finding is consistent with the results of the studies conducted in Uganda (14, 15), Cameroon (17), and Ethiopia (33). Women who disclosed their HIV status were more likely to have no fear of negative consequences from their partners, such as divorce, and received support from their spouse for retention in care (38). In addition, disclosing their HIV status to partners may reduce discrimination and fear associated with accessing HIV care services, which may make their stay on ART care comfortable (15, 39). Therefore, health workers in the PMTCT unit should strengthen counseling services for women on the importance of disclosure of HIV status to sexual partners throughout their care.

The study identified viral load suppression status as another risk factor for attrition. The risk of attrition among women who had unsuppressed/unknown viral load status was 2.61 times higher than those who had suppressed viral load status. This finding aligns with the result of the study conducted at the University of Gondar (36). The possible explanation is that women with unsuppressed viral load status may discontinue care due to the fear of providers' reaction to high viral loads (3, 23). Conversely, women with known viral load status may receive more attention and follow-up care from the providers, leading to better retention in care compared to those with unknown viral load status. Failure to achieve viral suppression augments the possibility of transmitting HIV to an infant through breastfeeding and puts children at risk of perinatal infection. Therefore, special attention should be given to women with unknown and unsuppressed viral load status to achieve global and national targets of zero new infections among HIV-exposed infants (40).

This study had a larger sample size and covered a wider geographic area. However, it had certain limitations. First, women who were LTFU might have continued their care at other facilities without documented referral. This might result in overestimation of the incidence of attrition. Second, women who transferred out from care were not traced, and they were excluded from the analysis, which may lead to underestimation of the attrition rate. Third, the information was based on routinely recorded data, which could include measurement and recording errors due to the nature of secondary data.

## 5 Conclusion

In this study, the cumulative incidence of attrition among women after the implementation of DTG-based regimens was found to be optimal. In addition, the risk of attrition from PMTCT care among women on DTG-based regimens is lower compared to those on EFV-based regimens. Poor adherence, non-disclosure of HIV status to partner, delivery at home, and unsuppressed/unknown viral load status were identified as risk factors for attrition in the study area.

## Data availability statement

The original contributions presented in the study are included in the article/supplementary material, further inquiries can be directed to the corresponding authors.

## Author contributions

WF: Conceptualization, Data curation, Formal analysis, Funding acquisition, Investigation, Methodology, Project administration, Resources, Software, Supervision, Validation, Visualization, Writing – original draft, Writing – review & editing. TT: Conceptualization, Data curation, Supervision, Validation, Writing – review & editing. EW: Conceptualization, Data curation, Supervision, Validation, Writing – review & editing. AA: Conceptualization, Data curation, Methodology, Software, Supervision, Validation, Visualization, Writing – review & editing.
